# Geographical variations in cancer mortality and social inequalities in southern Spain (Andalusia). 2002-2013

**DOI:** 10.1371/journal.pone.0233397

**Published:** 2020-05-22

**Authors:** Vanessa Santos-Sánchez, Juan Antonio Córdoba-Doña, Francisco Viciana, Antonio Escolar-Pujolar, Lucia Pozzi, Rebeca Ramis

**Affiliations:** 1 Department of Economics and Business, University of Sassari, Sassari, Italy; 2 Department of Preventive Medicine and Public Health, Jerez University Hospital, Jerez de la Frontera, Spain; 3 Institute of Statistics and Cartography of Andalusia, Seville, Spain; 4 Consejería de Salud de la Junta de Andalucía, Cádiz, Cádiz, Spain; 5 Cancer and Environmental Epidemiology Unit, National Center for Epidemiology, Carlos III Institute of Health, Madrid, Spain; 6 Center for Biomedical Research in Epidemiology & Public Health (CIBER Epidemiología y Salud Pública—CIBERESP), Madrid, Spain; University of California San Francisco, UNITED STATES

## Abstract

**Introduction:**

Geographical variations in cancer mortality can be explained, in part, by their association with social inequalities. The objective of our study was to analyse the spatial pattern of mortality in relation to the most common causes of cancer in the Spanish autonomous community of Andalusia and its possible association with social inequalities.

**Materials and methods:**

A small area cross-sectional study in Andalusia, with census tracts as units of spatial analysis, for the period 2002–2013. Cases and person-years, sex and age group came from the Longitudinal Population Database of Andalusia. Standardized mortality rates and smoothed risk ratios were calculated using the Besag, York and Mollié model for lung, colorectal, breast, prostate, bladder and stomach cancer. In order to evaluate the association with social inequalities we included the deprivation index of the census tract as a covariate.

**Results:**

The results show an East-West mortality pattern with higher risk in the west for lung and bladder cancer among men, and breast cancer among women. For all of Andalusia, the association between deprivation index of the census tract and mortality relative risks is positive and significant for lung, stomach and bladder cancers in men, while in women we observed a negative association for lung cancer and a positive for stomach cancer.

**Conclusions:**

Knowledge regarding the spatial distribution of cancer mortality and the socioeconomic inequalities related should contribute to the design of specific health and social policies–aimed at tackling cancer mortality and social inequalities in areas of high mortality and/or levels of deprivation.

## 1. Introduction

Cancer is one of the principal causes of death in Spain, occupying second place in terms of total deaths. Tumours have been the most common cause of death in men since 2005, with 295.20 per 100,000 in 2013, and the second most common cause of death for women, with 183.10 deaths per 100,000 in 2013 [1]. Compared with the rest of Europe/Europe overall, cancer mortality for men in Spain was higher for cancers of the stomach, large intestine, liver, larynx and bladder, while mortality in women was second lowest on the list, with the exception of stomach cancer, which was above the European average [2].

With respect to geographical distribution, cancer mortality data in Spain has presented a differential mortality pattern since the 1990s, with higher mortality rates in the south-west of the country. In the following decades, however, there has been a progressive convergence of the mortality figures of the municipalities of the south with the Spanish average [3].

Spain has a National Health System, which during the period 1981–2002 suffered a process of devolution of health care to the 17 autonomous communities that conform the country. This resulted in a decentralized health system with universal coverage for primary, specialized and hospital care, with minimal territorial differences in access to health care services [4].Andalusia, situated in the south of -Spain, is the second largest autonomous community by area, occupying 17.25% of Spanish territory. In addition, it is the fourth most populated region in Europe, and the most populous in Spain, with 8.50 million inhabitants. Administratively, the region is divided into eight provinces: Almeria, Cadiz, Cordoba, Granada, Huelva, Jaen, Malaga and Seville. According to the National Institute of Statistics, Andalusia is one of the autonomous communities in the country with highest risk of poverty, highest unemployment rates and lowest GDPs and health budgets per capita. A total 74% of Andalusian municipalities are at the highest levels of deprivation, with the most pronounced socio-economic inequalities being found in eastern Andalusia [5].

In a European context, cancer mortality in Andalusia is somewhere near the mean for both sexes, although lung cancer mortality is higher than the European average (75.80 per 100,000 versus 68.32 per 100,000), but lower for breast cancer (21.20 per 100,000 versus 28.28 per 100,000) [6].

In relation to Spain overall, Andalusia sits higher than the adjusted mean mortality rate in men, and has similar values to the mean in women. In 2013 in Andalusia, cancer was the most frequent cause of death in the 15–64 year-old age group, and the second most frequent for over 65s, preceded only by circulatory diseases [7]. In men, lung cancer was responsible for the highest cancer-related mortality rates and in women, breast cancer, although colorectal cancer has been the most frequent cause for both sexes [2].

The distribution of mortality by province across the region is similar for both sexes, presenting an East-West pattern with higher cancer mortality rates than the Andalusian average in the western provinces [3].

Knowledge about geographical patterns of mortality allows health policies to be targeted at the areas of highest risk. For that reason, in the last few decades there has been an increase in the number of studies that consider geographical area as a determinant of health, thanks to advances in Bayesian modelling as well as in Geographical Information Systems (GIS). These analyses permit not only the knowledge to be obtained about the detailed distribution of mortality, but also the study of the possible association of this distribution with other kinds of variables–as, in addition to other kinds of individual factors, there are contextual factors related to the geographical area that explain, at least in part, the health levels of the population.

In this context, many diverse small area ecological studies have been carried out that show an association between different social, behavioural or environmental risk factors and the incidence of, and mortality due to, cancer [8,9]. The association between cancer mortality and socioeconomic variables is a well-documented fact in the literature, showing in general terms that mortality and incidence increase in the population groups with the lowest socioeconomic levels [10].

The identification of geographical areas with higher cancer mortality and higher levels of deprivation facilitates an evaluation of the policies implemented to combat cancer–like the planning of interventions aimed at reducing inequality; despite this, in Spain these have only been dealt with by a number of studies focused on a limited range of cities at census-tract level [11,12]. Our article is the first time longitudinal mortality data for all of Andalusia–including the socioeconomic deprivation of the census tract, the smallest administrative unit for which mortality data is available–has been studied. Thus, the objectives of our study were to find the spatial pattern of mortality for the most frequent causes of cancer in Andalusia, as well as its possible association with the deprivation index of the census tract in the period 2002–2013.

## 2. Materials and methods

### 2.1. Data sources and variables

We carried out a cross-sectional small area study with the census tract as the spatial analysis unit. The study is comprised of the 5,381 census tracts that existed in the year 2001.

The data were obtained from the Longitudinal Population Database of Andalusia (BDLPA) [13] created by the Statistics and Cartography Institute of Andalusia (IECA) in 2002 and covered deaths from the selected causes, as well as the person-years, both by census tract, sex and age group (19 five-year groups, from 5 to 95 years old). The BDLPA combines data from the Housing and Population Census of Andalusia from 2001; the events recorded in the Natural Movement of the Population Database (MNP) e.g. deaths, births and marriages; and the changes to the register of inhabitants that have occurred since 2002. The starting population were those registered in the population and housing database in 2001 (7,357,547), who could be located in a council registry (7,202,794, 97.90% of those in the census), and who resided in Andalusia on the 1^st^ of January 2002. During the study period changes to residential or vital status were monitored through changes to council registers and via the death registry. An end to monitoring could be triggered by (i) death registered in the MNP (ii) emigration outside of Andalusia (iii) censure due to the end of the study on December 31^st^ 2013. Each case was assigned the census tract from 2001 that corresponded with the registered address at time of death.

The locations of tumours analysed for each sex, along with the corresponding code from the International Classification of Diseases (10^th^ rev.), were: malignant neoplasm of stomach (C16), malignant neoplasm of colon, rectum and anus (C18–C21), malignant neoplasm of trachea, bronchus and lung (C33–C34), malignant neoplasms of the breast in women (C50), malignant neoplasms of the prostate (C61) and malignant neoplasms of the bladder (C67).

In order to evaluate the magnitude of the association between social inequality and the risk of death from cancer, we have used the deprivation index by census tract for all of Andalusia [14]. Small area deprivation indices have been shown to be independently related to cancer mortality rates [15]. The index was worked out from the corresponding data from the Housing and Population Census of Andalusia in relation to the following: (i) percentage of persons with low education levels (ii) unemployment rate, and (iii) percentage of workers without qualifications. These variables belong to the 2001 census, in order to take into account the latency period of cancer, generally 10 years for solid tumours [16]. Through a principal component analysis, we constructed an index that classified the census tracts into five levels of deprivation, in accordance with the quintiles of the respective factorial ratings. The tracts with the lowest levels of deprivation were assigned Level 1, those with the highest, Level 5.

### 2.2. Statistical analysis

Standard mortality ratios (SMR) were calculated as the ratio between the observed cases and those expected by sex and cause. To calculate the number of expected cases, the total mortality rates for age group, sex and cause are multiplied by person-years per census tract for each age group and sex. We also calculated the 95% credibility intervals of the SMRs.

Following this, we calculated the smoothed relative risks (RRs) and their corresponding 95% credibility intervals. The RR determines whether an area has an equal (RR = 1), higher (RR>1) or lower (RR<1) occurrence of cases than that expected from the reference rates. The RRs were calculated via a Besag, York and Mollié generalized linear mixed model (GLMM) [17]. This model adjusts a spatial Poisson model with two types of random effects, an unstructured effect which accounts for unstructured heterogeneity, and a structured effect, the spatial term, which considers the contiguity between areas. In order to define the contiguity in between areas we have used the borders of the adjacent census tracts. The model used to analyse the geographical distribution of mortality is defined as follows:

$$O_{i}\sim Po\left(E_{i}\lambda_{i}\right)$$

$$\eta_{i}=\text{log}\left(\lambda_{i}\right)=\alpha +h_{i}+b_{i}$$ (1)

Where O_i_ denotes the cases observed in the census tract i, *E*_*i*_ are the expected cases, *λ*_*i*_ is the expected death rate, *α* is the intercept, *h*_*i*_ is the non-spatial random effect y *b*_*i*_ the spatial random effect. The non-spatial random effect (heterogeneity) is assumed to be a normal distribution with a zero mean and constant variance. For the random effect that captures the spatial variability, we have used an intrinsic conditional autoregressive model (CAR) [18,19].

In order to analyse the association of deprivation with cancer mortality, the census tract deprivation index has been included as an explanatory variable in the model, taking the census tracts with the lowest deprivation as reference categories. As such, the model takes the following form:

$$\eta_{i}=\text{log}\left(\lambda_{i}\right)=\alpha +h_{i}+b_{i}+\beta x_{i}$$ (2)

Where *e*^*β*^ is the RR associated with the deprivation index.

Using Besag, York and Mollié models with explanatory variables, we have analysed the association of the deprivation index with all of the studied forms of cancer across all of Andalusia. We also used this model to analyse each of the eight Andalusian province capitals individually in order to evaluate the risk within every city separately.

To determine the goodness of fit of the models we have utilized the deviance information criterion (DIC), a generalization of the Akaike information criterion (AIC) [20], in which the models with lower DIC will provide better adjustment.

The tool used for Bayesian inference of the subsequent marginal distributions for models parameters was Integrated Nested Laplace Approximations (INLA) [21]. For this, we used the R-INLA library version 18.07.12 [22] available in the R statistical package version 3.6.0 [23].

In order to produce the maps, smooth RRs were divided into 9 classes following quantiles, with the aim of guaranteeing the homogeneity of all of the geographical areas and be able to interpret the geographical distribution of the RRs properly [24]. For this we used version 10.5 of the ArcMap software package.

## 3. Results

### General

From 2002 to 2013 in the BDLPA a total of 337,561 deaths were registered for all forms of cancer in the age groups analysed, 214,863 (63.65%) in men and 122,698 (36.35%) in women. The total person-years for the period and age groups studied was 75,678,680, of which 37,004,773 (48.90%) were men-years and 38,673,907 (51.10%) were women-years.

**Fig 1:**
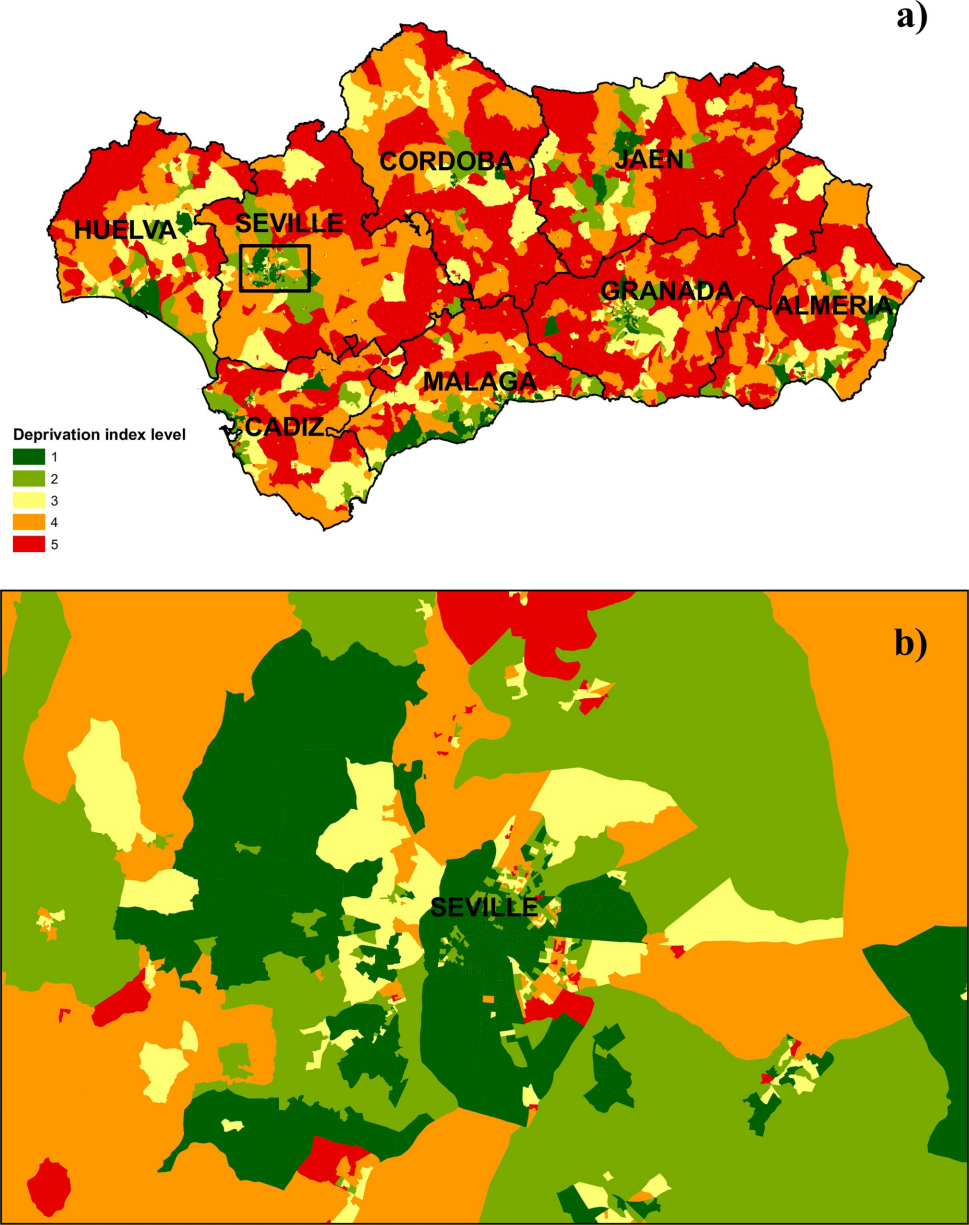
Spatial distribution of deprivation index by census tract for: a) Andalusia overall, b) large scale inset map showing the city of Seville.

Fig 1A shows the spatial distribution by census tract of the five categories in the deprivation index analysed in the study for all eight of the Andalusian provinces (5,381 census tracts), as well as a large scale inset map showing the city of Seville (Fig 1B). A higher frequency of more deprived census tracts (level 5) can be seen in rural areas or those with scarce population, while in urban areas the distribution of deprivation levels is more heterogeneous.

**Fig 2:**
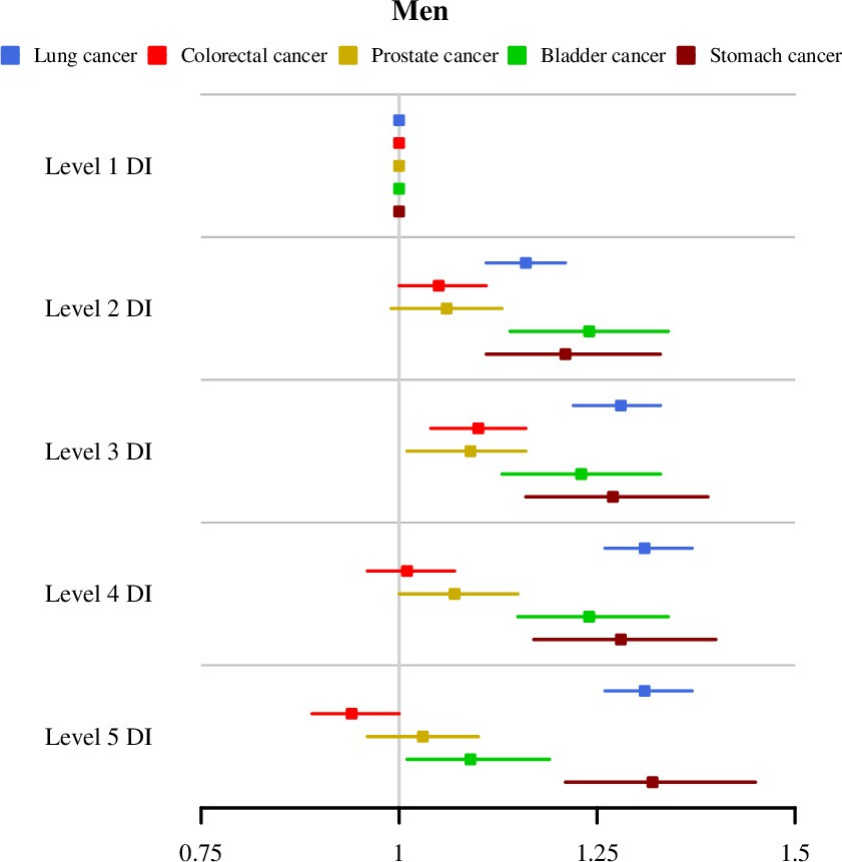
Mortality RRs and 95% credibility intervals by deprivation index level and type of cancer. All Andalusia. Men.

**Fig 3:**
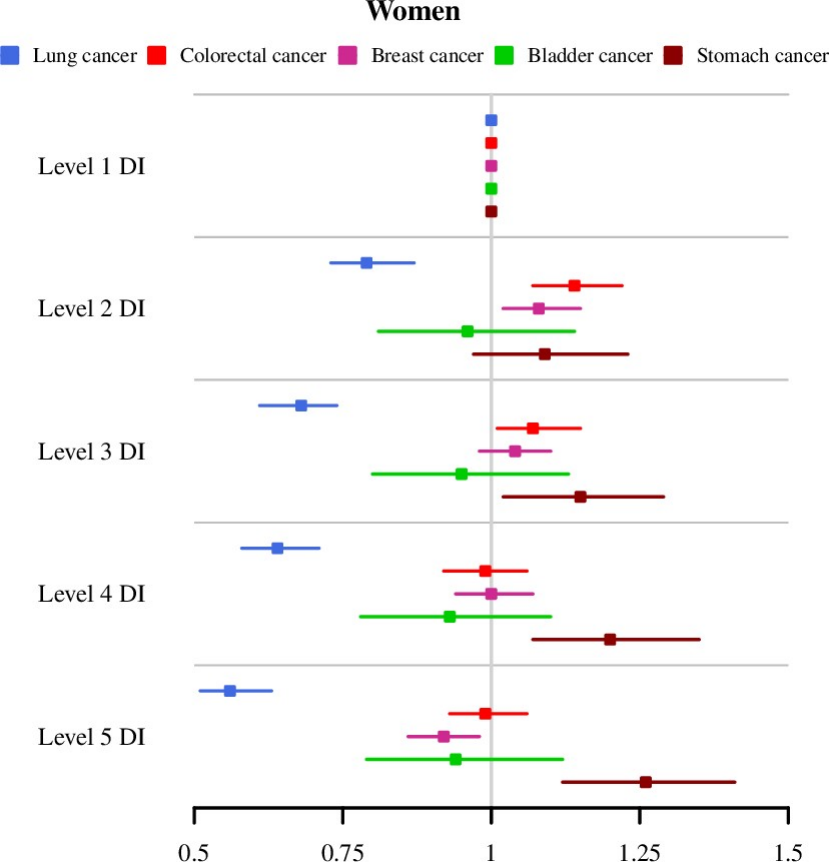
Mortality RRs and 95% credibility intervals by deprivation index level and type of cancer. All Andalusia. Women.

For all of Andalusia, all types of cancer studied showed differences in mortality RRs according to the deprivation index category (see Table 1 and Figs 2 and 3).

**Table 1:** Mortality RRs adjusted by deprivation index level, type of cancer and sex. All Andalusia.

		Men			Women		
Cause	DI	n	RR	CI 95%	n	RR	CI 95%
Lung cancer	1	5188	**1.00**	-	1168	**1.00**	-
	2	6013	**1.16**	1.11–1.21	895	**0.79**	0.73–0.87
	3	6750	**1.28**	1.22–1.33	747	**0.68**	0.61–0.74
	4	6942	**1.31**	1.26–1.37	679	**0.64**	0.58–0.71
	5	6636	**1.31**	1.26–1.37	545	**0.56**	0.51–0.63
Colorectal cancer	1	2256	**1.00**	-	1799	**1.00**	-
	2	2724	**1.05**	1.00–1.11	2048	**1.14**	1.07–1.22
	3	2950	**1.10**	1.04–1.16	1937	**1.07**	1.01–1.15
	4	2769	1.01	0.96–1.07	1769	0.99	0.92–1.06
	5	2513	0.94	0.89–1.00	1652	0.99	0.93–1.06
Breast cancer	1	-	-	-	2265	**1.00**	-
	2	-	-	-	2370	**1.08**	1.02–1.15
	3	-	-	-	2213	1.04	0.98–1.10
	4	-	-	-	2050	1.00	0.94–1.07
	5	-	-	-	1707	**0.92**	0.86–0.98
Prostate cancer	1	1569	**1.00**		-	-	-
	2	1710	1.06	0.99–1.13	-	-	-
	3	1868	**1.09**	1.01–1.16	-	-	-
	4	1939	**1.07**	1.00–1.15	-	-	-
	5	1871	1.03	0.96–1.10	-	-	-
Bladder cancer	1	1170	**1.00**	-	272	**1.00**	-
	2	1481	**1.24**	1.14–1.34	263	0.96	0.81–1.14
	3	1538	**1.23**	1.13–1.33	266	0.95	0.80–1.13
	4	1608	**1.24**	1.15–1.34	262	0.93	0.78–1.10
	5	1398	**1.09**	1.01–1.19	250	0.94	0.79–1.12
Stomach cancer	1	869	**1.00**	-	560	**1.00**	-
	2	1062	**1.21**	1.11–1.33	607	1.09	0.97–1.23
	3	1140	**1.27**	1.16–1.39	637	**1.15**	1.02–1.29
	4	1161	**1.28**	1.17–1.40	659	**1.20**	1.07–1.35
	5	1160	**1.32**	1.21–1.45	640	**1.26**	1.12–1.41

DI: Deprivation index level; n: number of observed cancer deaths; RR: Relative risks; CI 95%: Credibility interval. Reference category: Level 1 of DI (less deprivation). Bold: Statistically significant result

In order to evaluate the fit of the models, we have calculated the DIC and the number of effective parameters of the models both with and without the covariable (Table 2). For all cancers examined, except prostate cancer and bladder cancer in women, we observed a low DIC and thus a better fit for the models which included the deprivation index.

**Table 2:** Deviances comparisons of the models with and without deprivation index by sex and cause.

	Men				Women			
Cause	DIC	PEFF	DIC PI	PEFF PI	DIC	PEFF	DIC PI	PEFF PI
Lung cancer	25,725.69	1,476.66	25,550.07	1,312.63	12,156.94	398.49	12,011.40	218.56
Colorectal cancer	19,685.08	313.85	19,659.88	286.55	17,176.21	228.82	17,155.06	207.21
Breast cancer	-	-	-	-	18,181.21	373.42	18,163.59	349.44
Prostate cancer	16,801.16	116.09	16,802.05	118.34	-	-	-	-
Bladder cancer	15,830.04	445.30	15,793.43	411.80	6,359.43	26.79	6,366.69	31.09
Stomach cancer	13,878.05	135.12	13,838.33	109.70	10,686.98	152.26	10,676.35	138.52

DIC: Deviance information criterion; PEFF: Number of effective parameters; DI: Deprivation index.

Table 3 shows the distribution of the mortality RRs adjusted by deprivation index for the eight Andalusian province capitals.

**Table 3:** Mortality RRs adjusted by deprivation index level, type of cancer and sex. Province capitals.

				Lung cancer						Colorectal cancer						Breast cancer			Prostate cancer			Bladder cancer						Stomach cancer				
				Men			Women			Men			Women			Women			Men			Men			Women			Men			Women	
Municipality	DI	CT	n	RR	CI 95%	n	RR	CI 95%	n	RR	CI 95%	n	RR	CI 95%	n	RR	CI 95%	n	RR	CI 95%	n	RR	CI 95%	n	RR	CI 95%	n	RR	CI 95%	n	RR	CI 95%
Almeria	1	46	248	1.00	-	61	1.00	-	129	1.00	-	87	1.00	-	112	1.00	-	80	1.00	-	66	1.00	-	15	1.00	-	30	1.00	-	28	1.00	-
	2	31	191	0.93	0.75–1.15	38	0.90	0.59–1.38	100	0.99	0.74–1.32	97	**1.47**	1.07–2.00	89	0.99	0.73–1.34	61	0.92	0.64–1.33	58	1.23	0.84–1.79	14	1.01	0.45–2.25	36	1.27	0.74–2.16	24	1.14	0.64–2.01
	3	20	164	**1.36**	1.08–1.71	20	0.63	0.34–1.17	76	1.12	0.80–1.56	56	1.33	0.90–1.96	51	0.92	0.62–1.36	51	1.13	0.75–1.71	51	**1.54**	1.02–2.34	6	0.78	0.26–2.36	32	1.64	0.91–2.96	16	1.05	0.51–2.17
	4	10	135	1.22	0.89–1.67	14	1.02	0.51–2.03	60	0.98	0.61–1.58	25	0.80	0.42–1.51	44	0.66	0.36–1.21	37	1.08	0.61–1.92	37	1.38	0.77–2.45	1	0.42	0.06–3.23	20	**2.11**	1.05–4.25	15	1.76	0.80–3.90
	5	11	97	**1.91**	1.43–2.54	7	0.48	0.17–1.34	37	1.38	0.87–2.18	29	1.46	0.86–2.48	23	1.05	0.61–1.78	14	0.61	0.26–1.40	18	1.17	0.59–2.32	8	2.47	0.89–6.85	27	**3.40**	1.79–6.47	7	0.78	0.24–2.59
Cadiz	1	34	185	1.00	-	40	1.00	-	84	1.00	-	64	1.00	-	78	1.00	-	41	1.00	-	62	1.00	-	11	1.00	-	34	1.00	-	23	1.00	-
	2	23	169	**1.26**	1.01–1.57	17	0.59	0.33–1.05	65	1.05	0.75–1.46	55	1.07	0.73–1.55	62	1.11	0.79–1.56	34	1.04	0.65–1.66	42	0.90	0.60–1.35	12	1.34	0.59–3.08	35	1.40	0.86–2.27	16	0.91	0.48–1.73
	3	29	236	**1.43**	1.16–1.76	22	0.67	0.39–1.15	85	1.08	0.78–1.48	66	1.14	0.80–1.64	66	0.96	0.68–1.35	49	1.22	0.79–1.90	55	0.89	0.60–1.31	7	0.65	0.24–1.78	43	1.47	0.92–2.34	30	1.55	0.89–2.70
	4	19	177	**1.61**	1.28–2.03	17	0.65	0.34–1.24	69	1.29	0.91–1.84	48	1.25	0.84–1.87	51	1.11	0.75–1.64	33	0.75	0.42–1.34	46	1.21	0.80–1.83	8	1.31	0.52–3.29	27	1.29	0.74–2.24	16	1.19	0.61–2.32
	5	6	171	**1.98**	1.42–2.78	12	1.13	0.47–2.69	65	**1.67**	1.01–2.77	38	1.41	0.78–2.56	25	1.06	0.56–2.02	41	1.38	0.66–2.88	29	1.63	0.92–2.91	12	2.16	0.68–6.92	20	1.08	0.42–2.79	10	1.14	0.39–3.34
Cordoba	1	64	331	1.00	-	54	1.00	-	173	1.00	-	115	1.00	-	140	1.00	-	99	1.00	-	83	1.00	-	18	1.00	-	46	1.00	-	36	1.00	-
	2	61	375	1.12	0.95–1.31	43	0.87	0.57–1.33	204	1.11	0.89–1.38	130	1.11	0.85–1.44	155	1.15	0.90–1.47	98	0.88	0.65–1.20	84	1.06	0.77–1.45	17	0.83	0.41–1.67	64	1.44	0.95–2.20	29	0.81	0.48–1.36
	3	45	396	**1.24**	1.05–1.47	40	0.70	0.44–1.14	190	1.09	0.87–1.37	105	0.98	0.74–1.31	130	0.95	0.73–1.25	98	0.92	0.68–1.26	74	0.88	0.62–1.25	22	1.11	0.56–2.18	60	1.45	0.93–2.24	32	0.90	0.53–1.53
	4	34	310	**1.34**	1.12–1.60	26	0.85	0.51–1.40	127	1.02	0.79–1.31	87	1.09	0.80–1.47	109	1.23	0.94–1.63	79	0.85	0.60–1.20	84	**1.42**	1.02–1.97	21	1.63	0.84–3.14	44	**1.63**	1.03–2.58	22	0.90	0.50–1.61
	5	20	179	**1.53**	1.23–1.90	19	1.24	0.69–2.23	71	1.23	0.91–1.68	46	1.16	0.78–1.71	48	1.18	0.81–1.71	37	0.89	0.57–1.39	45	1.49	0.99–2.26	5	0.82	0.27–2.43	21	1.54	0.85–2.77	13	1.01	0.48–2.13
Granada	1	94	400	1.00	-	120	1.00	-	223	1.00	-	189	1.00	-	178	1.00	-	174	1.00	-	87	1.00	-	28	1.00	-	66	1.00	-	52	1.00	-
	2	40	226	1.11	0.93–1.33	44	0.77	0.53–1.12	141	**1.31**	1.04–1.66	104	1.19	0.92–1.54	102	**1.32**	1.02–1.71	85	0.92	0.68–1.23	64	**1.63**	1.16–2.30	15	1.12	0.57–2.17	48	1.42	0.95–2.13	23	1.09	0.66–1.80
	3	24	163	**1.28**	1.04–1.57	23	0.69	0.43–1.12	95	**1.48**	1.13–1.93	73	1.31	0.96–1.77	54	1.05	0.75–1.48	57	0.99	0.69–1.41	50	**2.10**	1.44–3.07	10	1.04	0.45–2.40	28	1.49	0.93–2.40	14	1.07	0.58–1.99
	4	11	63	**1.49**	1.11–1.99	8	0.59	0.27–1.29	33	1.40	0.94–2.10	18	0.98	0.59–1.60	14	0.64	0.35–1.20	31	1.38	0.86–2.19	15	1.03	0.50–2.15	6	1.42	0.49–4.12	10	1.47	0.72–2.99	6	0.99	0.39–2.51
	5	12	67	**1.81**	1.38–2.39	7	0.66	0.30–1.44	25	1.27	0.83–1.97	22	1.32	0.83–2.09	21	1.30	0.81–2.09	26	1.57	0.99–2.49	16	**1.87**	1.03–3.38	1	0.42	0.06–3.09	11	**1.94**	1.01–3.72	11	**2.50**	1.28–4.85
Huelva	1	40	214	1.00	-	46	1.00	-	91	1.00	-	68	1.00	-	94	1.00	-	65	1.00	-	54	1.00	-	11	1.00	-	42	1.00	-	21	1.00	-
	2	31	214	1.21	0.99–1.47	26	0.77	0.47–1.25	83	1.14	0.84–1.56	63	1.23	0.86–1.76	73	1.00	0.73–1.38	48	0.85	0.58–1.26	48	1.02	0.68–1.54	10	1.24	0.52–2.94	47	1.37	0.90–2.10	24	1.53	0.84–2.76
	3	10	116	**1.38**	1.05–1.83	21	1.41	0.78–2.57	53	1.39	0.91–2.13	26	1.24	0.73–2.10	34	1.14	0.72–1.80	25	0.85	0.46–1.57	24	1.31	0.75–2.28	7	1.64	0.52–5.20	11	0.77	0.36–1.65	6	0.83	0.28–2.43
	4	15	167	**1.92**	1.53–2.41	15	0.85	0.46–1.60	57	**1.50**	1.04–2.17	44	**1.60**	1.06–2.41	46	1.14	0.76–1.70	29	0.85	0.51–1.41	40	**1.66**	1.06–2.60	5	1.19	0.41–3.47	23	1.46	0.86–2.48	18	1.70	0.84–3.42
	5	5	99	**2.00**	1.39–2.87	11	1.86	0.82–4.19	35	**1.85**	1.05–3.26	24	1.91	0.98–3.70	19	1.14	0.56–2.30	19	0.84	0.33–2.11	10	0.82	0.29–2.29	4	1.06	0.14–8.33	17	1.58	0.66–3.77	8	**3.35**	1.33–8.45
Jaen	1	29	127	1.00	-	22	1.00	-	74	1.00	-	66	1.00	-	67	1.00	-	62	1.00	-	36	1.00	-	6	1.00	-	30	1.00	-	13	1.00	-
	2	20	91	1.06	0.78–1.44	13	0.73	0.34–1.55	56	1.07	0.73–1.56	42	0.84	0.55–1.28	46	0.87	0.57–1.32	47	1.02	0.68–1.53	30	1.12	0.67–1.87	6	1.22	0.37–4.02	28	1.23	0.71–2.13	21	**2.34**	1.16–4.74
	3	13	97	1.33	0.96–1.83	11	0.78	0.33–1.83	41	1.05	0.69–1.61	30	0.98	0.61–1.56	30	0.92	0.57–1.48	29	0.88	0.54–1.41	27	1.14	0.65–2.01	1	0.39	0.05–3.28	19	1.40	0.78–2.51	24	**2.38**	1.09–5.18
	4	12	108	1.28	0.90–1.82	13	1.15	0.51–2.61	58	1.38	0.90–2.11	28	0.83	0.48–1.43	32	0.53	0.27–1.00	35	0.89	0.52–1.51	27	1.56	0.88–2.74	4	0.51	0.06–4.26	21	1.28	0.66–2.48	12	1.42	0.53–3.76
	5	2	33	1.23	0.54–2.81	6	2.13	0.49–9.25	18	0.63	0.15–2.61	13	0.38	0.05–2.77	11	1.07	0.33–3.49	10	1.46	0.52–4.11	5	-	-	3	-	-	8	0.73	0.10–5.43	7	**5.47**	1.52–9.66
Malaga	1	140	646	1.00	-	158	1.00	-	281	1.00	-	202	1.00	-	252	1.00	-	157	1.00	-	118	1.00	-	22	1.00	-	85	1.00	-	55	1.00	-
	2	129	723	**1.15**	1.03–1.29	112	**0.74**	0.58–0.96	290	1.06	0.90–1.25	246	**1.25**	1.04–1.51	313	**1.31**	1.11–1.56	188	1.20	0.97–1.48	177	**1.49**	1.17–1.89	31	1.44	0.83–2.49	100	1.21	0.90–1.62	56	1.06	0.72–1.54
	3	92	588	**1.28**	1.14–1.44	88	0.81	0.62–1.06	254	**1.24**	1.04–1.47	165	1.10	0.89–1.36	196	1.13	0.93–1.38	146	1.21	0.96–1.52	134	**1.52**	1.18–1.95	25	1.51	0.85–2.68	94	**1.57**	1.17–2.11	47	1.18	0.80–1.75
	4	47	324	**1.40**	1.21–1.62	36	0.75	0.52–1.09	109	0.99	0.78–1.26	80	1.17	0.89–1.53	107	**1.32**	1.03–1.68	59	0.99	0.72–1.36	67	**1.50**	1.09–2.06	15	1.90	0.95–3.78	56	**1.70**	1.19–2.43	26	1.00	0.58–1.74
	5	14	110	**2.04**	1.62–2.56	14	1.15	0.64–2.05	38	1.27	0.85–1.89	24	1.25	0.79–2.00	32	1.44	0.95–2.19	18	0.71	0.36–1.39	19	**1.74**	1.02–2.97	1	0.55	0.07–4.12	14	1.57	0.81–3.05	7	1.39	0.59–3.27
Seville	1	208	1069	1.00	-	270	1.00	-	548	1.00	-	412	1.00	-	538	1.00	-	330	1.00	-	260	1.00	-	77	1.00	-	170	1.00	-	122	1.00	-
	2	124	748	**1.20**	1.08–1.32	129	0.84	0.68–1.04	363	**1.15**	1.00–1.32	298	**1.25**	1.08–1.46	305	1.01	0.87–1.16	203	1.06	0.88–1.27	206	**1.37**	1.13–1.65	38	0.87	0.59–1.29	99	1.03	0.80–1.32	83	1.30	0.97–1.73
	3	76	502	**1.26**	1.12–1.41	67	**0.69**	0.53–0.91	255	**1.23**	1.05–1.43	190	**1.24**	1.04–1.48	198	1.06	0.90–1.25	146	1.15	0.94–1.41	139	**1.41**	1.14–1.75	28	0.91	0.58–1.43	74	1.20	0.91–1.58	58	**1.43**	1.04–1.97
	4	66	514	**1.53**	1.37–1.72	75	0.95	0.73–1.24	222	**1.26**	1.07–1.48	141	1.09	0.89–1.33	165	1.06	0.88–1.27	148	**1.31**	1.07–1.61	125	**1.45**	1.16–1.81	17	0.73	0.43–1.23	66	1.18	0.88–1.58	40	1.17	0.81–1.69
	5	36	277	**1.97**	1.70–2.28	31	0.84	0.57–1.24	117	**1.58**	1.28–1.95	82	**1.36**	1.06–1.74	67	0.95	0.73–1.23	62	**1.38**	1.04–1.83	46	1.26	0.90–1.75	14	1.18	0.65–2.14	39	**1.72**	1.20–2.46	37	**2.43**	1.67–3.53

DI: Deprivation index level; CT: Number of census tracts; n: number of observed cancer deaths; RR: Relative risks; CI 95%: Credibility interval. Reference category: Level 1 of DI (less deprivation). Bold: Statistically significant result.

The following section presents the results by cancer type for each sex, ordered by the number of deaths caused by each type:

### Lung cancer

Deaths from lung cancer represented 10.54% of total cancer deaths in the cohort. A total of 35,563 fatalities were registered for this type, of which 31,529 (88.66%) were men and 4,034 (11.34%) were women.

**Fig 4:**
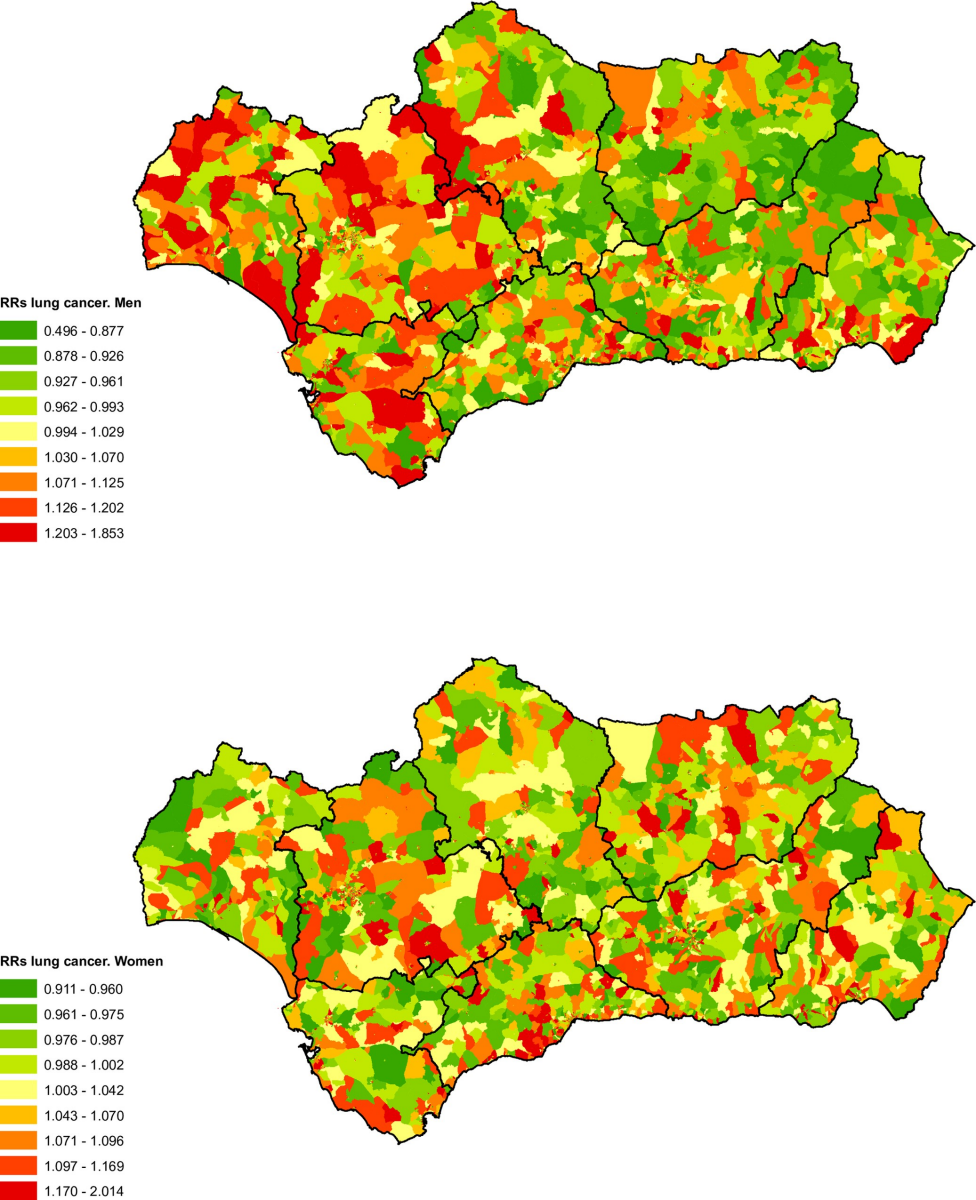
Spatial distribution of lung cancer mortality by census tract and sex.

Fig 4 show the spatial distribution maps for lung cancer RRs according to census tract for men and women. We noted that the geographical pattern is very different for men and for women. For the former a marked East-West pattern is evident, with higher risk in the western part, specifically in the provinces of Cadiz, Huelva and Seville. For women there was not a definite pattern, although cities like Seville present notably higher mortality from this type, with numerous high-risk census tracts.

We found a social gradient in the mortality RRs for all of Andalusia in both sexes, with positive RRs for men (higher mortality in groups with higher deprivation levels) but negative for women (Table 1).

For men this pattern was reproduced in all of the province cap itals with the exceptions of Almeria and Jaen, which did not present clear gradients, although for these municipalities we continued to observe an excess of risk in the categories related to the highest levels of deprivation, reaching 1.91 in Almeria (Table 3). For women we did not obtain statistically significant results for the capitals, probably as a result of the reduced number of cases.

### Cancers of the colon, rectum and anus

There were 22,717 deaths as a result of cancers of the colon, rectum and anus during the study period, of which 59.48% were men. These deaths represented 6.73% of the deaths from cancer recorded in the BDLPA.

**Fig 5:**
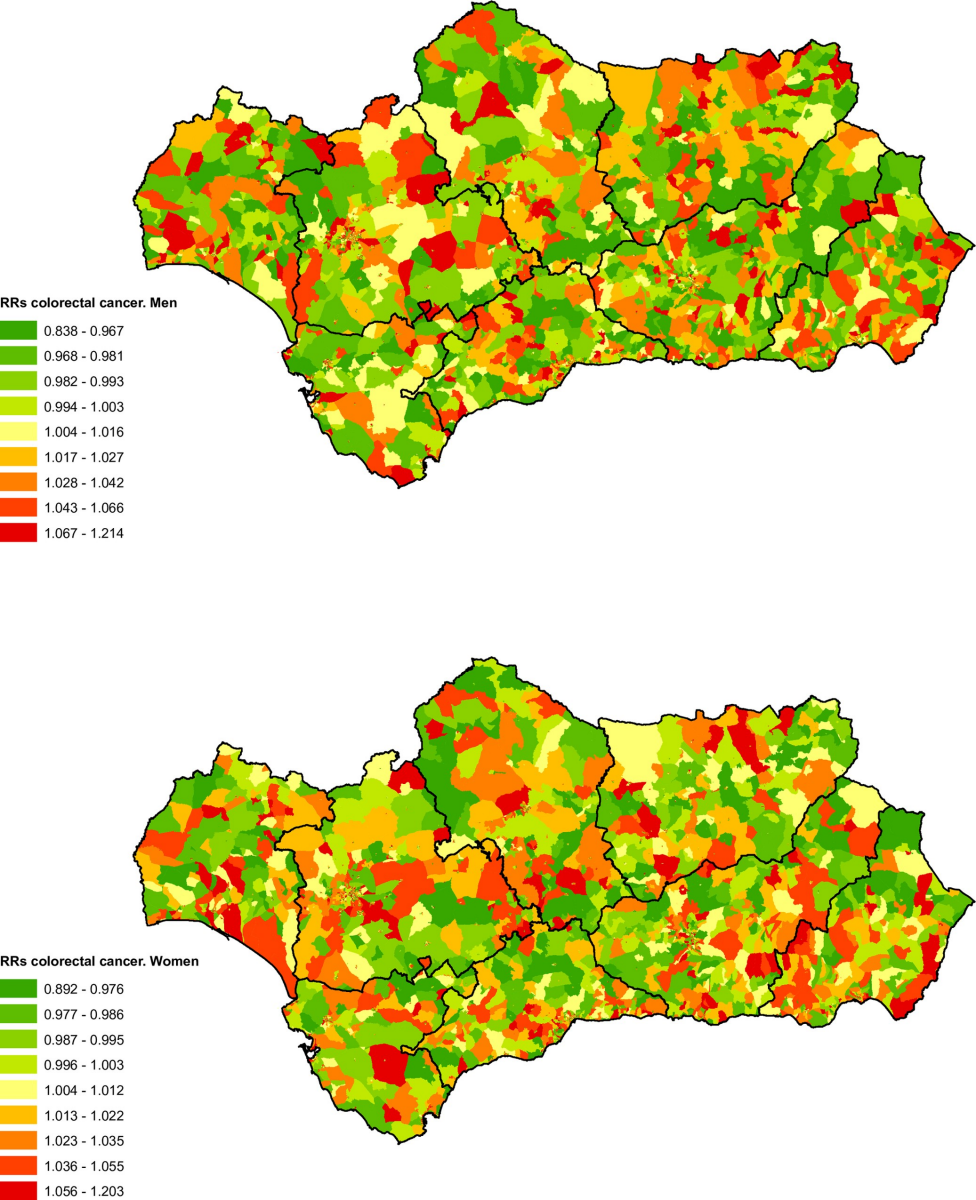
Spatial distribution of colorectal cancer mortality by census tract and sex.

Both in men and women the spatial distribution of mortality RRs associated with colorectal cancer did not show a notable pattern (Fig 5). The analysis of the deprivation-level adjusted RRs for all of Andalusia show an inverted U-shaped pattern for either sex. We observed a small increase in RRs for levels 2 and 3, while in the higher deprivation levels the RRs present values close to one, indicating a similar risk to the reference category.

We observed a positive social gradient for men in the city of Seville (Table 3). The category of highest deprivation showed a significant increase in mortality RRs in Cadiz and Huelva for men and in Seville for women.

### Breast cancer

There were a total of 10,605 deaths from breast cancer between 2002 and 2013 in our study. This number represented 8.64% of all deaths from cancer which occurred in the Andalusian cohort.

**Fig 6:**
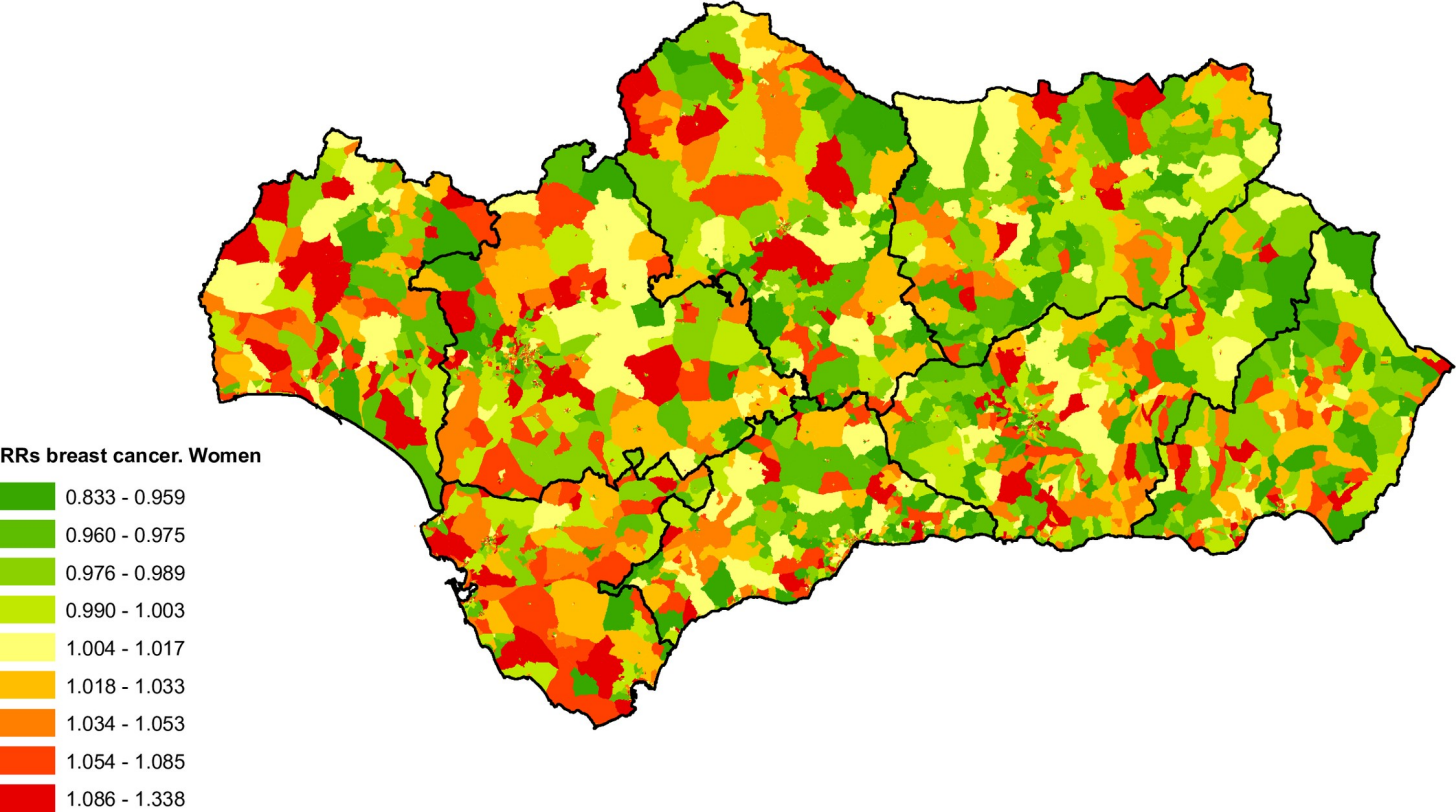
Spatial distribution of breast cancer mortality in women by census tract.

The spatial distribution map of the RRs for mortality due to breast cancer (Fig 6) shows a weak East-West pattern, with the western provinces of Cadiz, Huelva and Seville standing out due to a high number of census tracts with high mortality RRs.

As can be seen in Table 1, for all of Andalusia the adjusted mortality for deprivation level shows an increase in RRs for categories 2 and 3, while in the census tracts with the highest deprivation there is a decrease in mortality RRs for breast cancer. The results by provincial capitals do not show any clear trends in mortality RRs for this cause of death.

### Prostate cancer

Between 2002 and 2013 a total of 8,957 deaths from prostate cancer were recorded, representing 4.17% of the total cancer deaths for men recorded in the BDLPA.

**Fig 7:**
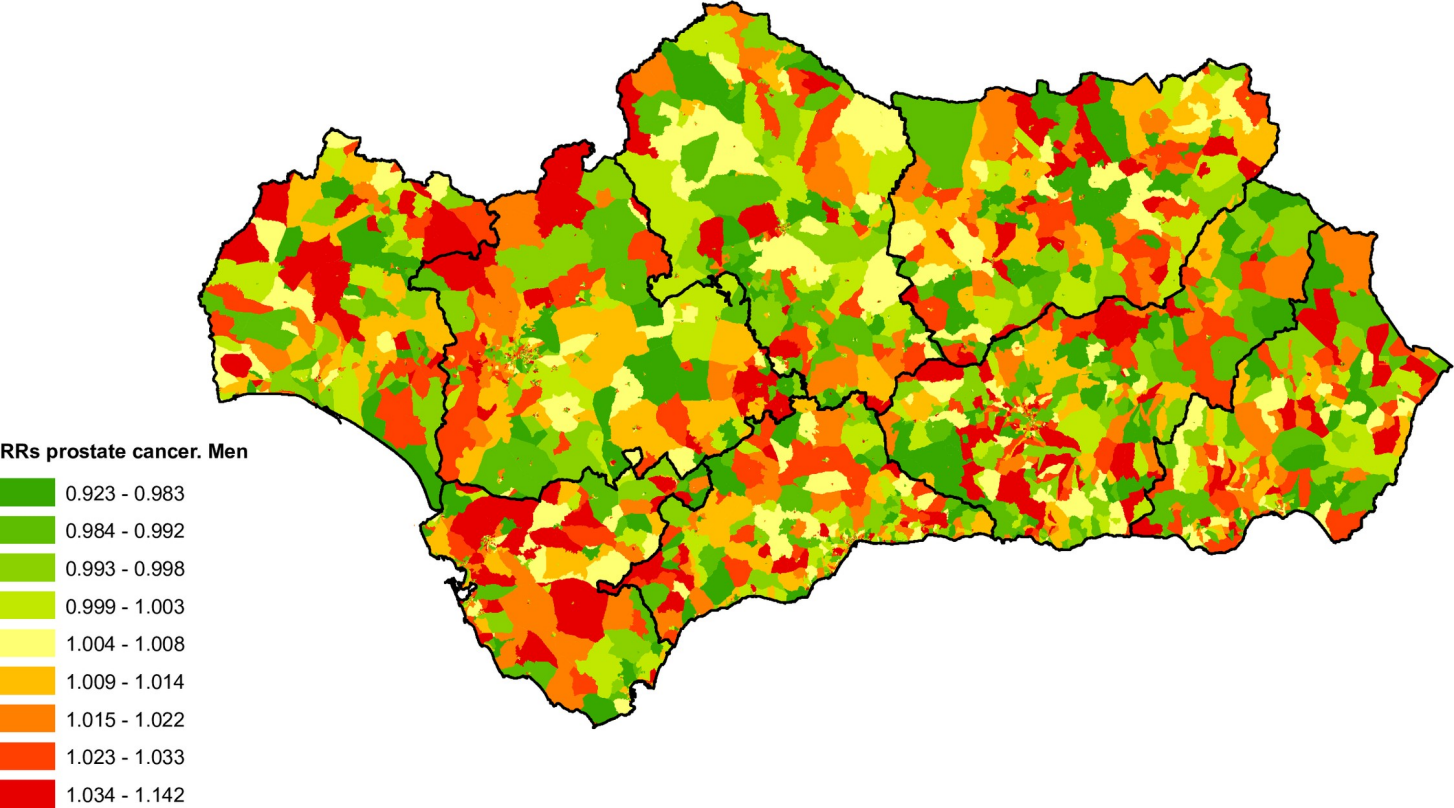
Spatial distribution of prostate cancer mortality by census tract.

Fig 7 shows the spatial pattern of mortality RRs for prostate cancer is far from clear. However, there is an excess of mortality in the province of Cadiz, as well as a large number of census tracts with low risk in the province of Cordoba.

We observed that for all of Andalusia the results of the mortality RRs analysis according to the deprivation index were not conclusive, although an inverted U-shaped pattern can be observed. Categories 3 and 4 showed a modest but statistically significant increase in the RRs for mortality (Table 1). With the exception of an increase in RRs for deprivation Levels 4 and 5 noted in Seville, the analysis of the capitals did not show significant results (Table 3).

### Bladder cancer

**Fig 8:**
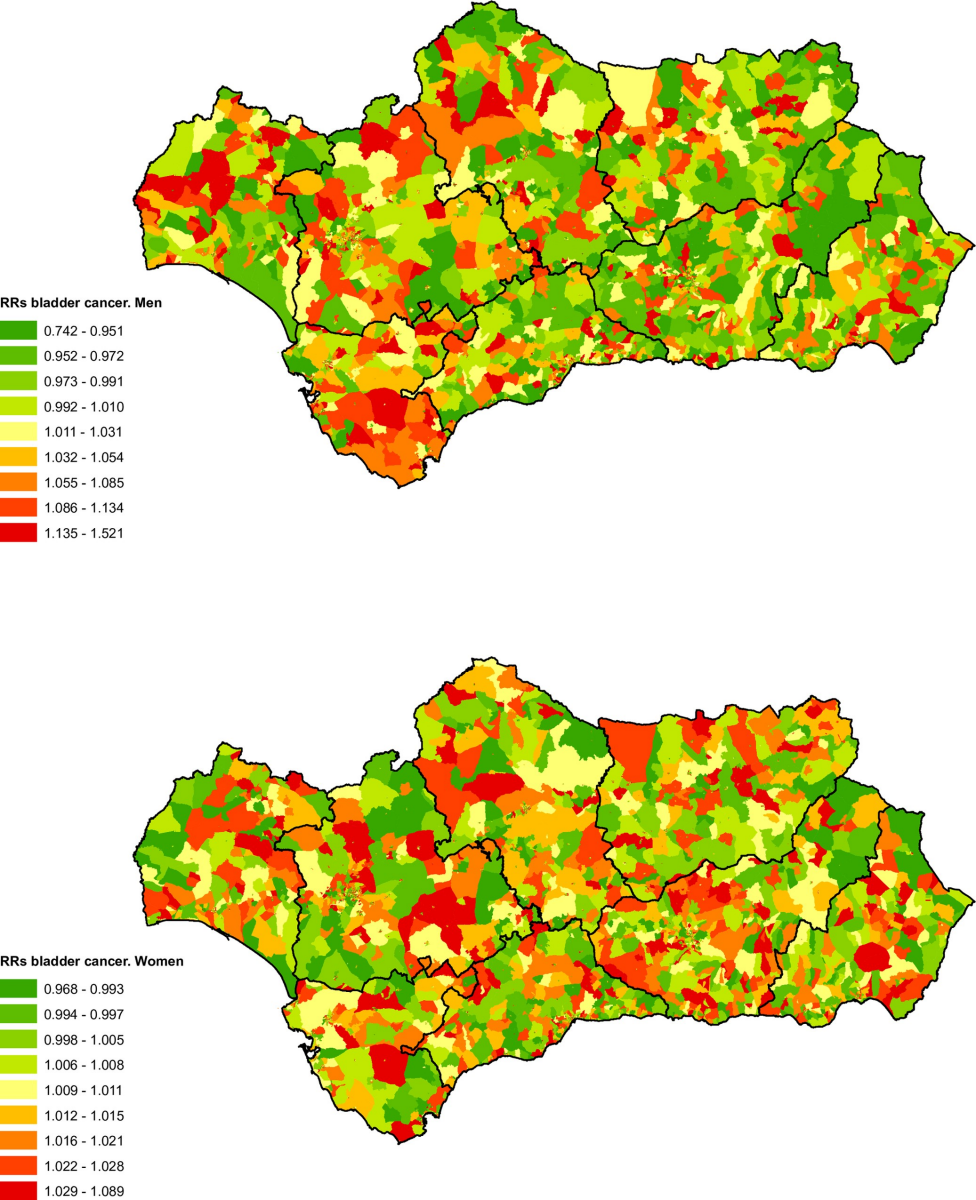
Spatial distribution of bladder cancer mortality by census tract and sex.

From 2002 until 2013 there were 8,508 deaths attributable to bladder cancer (2.52% of total deaths from tumours in the BDLPA), 7,195 in men (84.57%) and 1,313 in women (15.43%). For men the spatial distribution of bladder cancer mortality by census tract (Fig 8) shows a pattern with higher mortality in the western provinces, especially Cadiz. For women it was difficult to discern a clear geographical pattern, with risk uniformly distributed across the region (Fig 8).

Table 1 shows the adjusted RRs for bladder cancer mortality according to the deprivation index for all of Andalusia. There is an increase in risk for men according to census tract classification, with levels 2, 3 and 4 showing higher RRs. In women we observed the opposite, a moderate but not statistically significant reduction in RRs for the most deprived census tracts.

As shown in Table 3, the results of the analysis for provincial capitals highlight the positive social gradient observed in men in the city of Malaga (more deprivation, more risk), in Seville we found an association similar to that found for all of Andalusia. As a result of the reduced number of deaths in women due to bladder cancer, the RRs are not statistically significant in any of the municipalities analysed. For the same reason, it was not possible to calculate RRs and their corresponding 95% credibility intervals for Level 5 deprivation in the city of Jaen.

### Stomach cancer

From 2002 to 2013 the registry recorded a total of 8,495 deaths due to this cause, which represented 2.52% of the total deaths from malignant tumours in the cohort, 5,392 (63.47%) were in men and 3,103 were in women (36.53%).

**Fig 9:**
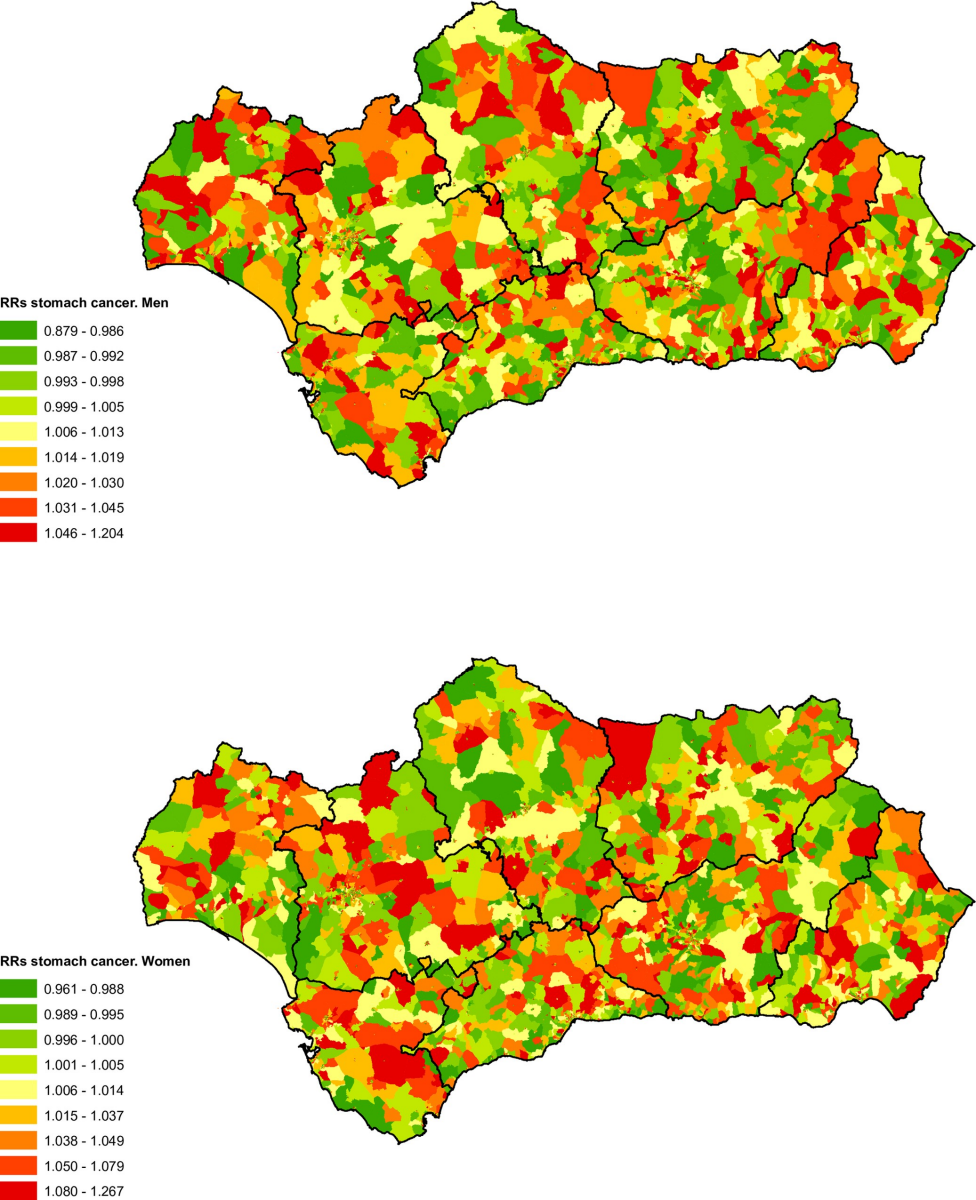
Spatial distribution of stomach cancer mortality by census tract and sex.

We did not find a clear spatial pattern for stomach cancer mortality in either sex (Fig 9), although the spatial distribution of RRs was similar for both. There was a positive social gradient for all of Andalusia for both sexes (Table 1), with statistically significant RRs for almost all levels of deprivation and somewhat higher RRs for men. We found a statistically significant excess of risk for men in the census tracts of greatest deprivation in Almeria, Granada and Seville. For women we obtained excess risk in the most deprived categories for Granada, Huelva, Jaen and Seville (Table 3).

## 4. Discussion

The results obtained from the analysis of 12 years of data from the BDLPA present an East-West pattern for lung and bladder cancer. For women, the same pattern presents itself for breast cancer. In addition, the association between the deprivation index of the census tract and the mortality RRs is both statistically significant and positive (higher levels of deprivation equal higher risk) for cancers of the lung, bladder, and stomach in men; for women, there was a negative social gradient for lung cancer, but a positive one for stomach cancer.

The reduction of socioeconomic inequalities in mortality found in the study requires investments in public health activities aimed at preventing disease. These activities include the development of health promotion initiatives, education on prevention measures or the monitoring of the population's health status. These actions could be more effective if they were intensified in areas of higher mortality and/or deprivation, so studies at the small area level would be useful in defining health policies in specific areas.

### Lung cancer

In Andalusia the 2013 mortality rate for lung cancer in men was 59.38 per 100,000 inhabitants, for women it was 9.24 per 100,000. These rates have experienced an increase in the last few years, which is actually more marked in women (4.20%) [2]. The geographical pattern for these tumours in men has not changed since the late 1990s, although there has been a slight reduction in the excess of risk [25]. This higher mortality has been attributed to the historically high levels of tobacco consumption in the Andalusian provinces, as well as socioeconomic, environmental and occupational factors [3]. The social gradients (positive for men and negative for women) were also observed by previous studies [26,27]. Taking into account that tobacco is responsible for 80% of cancers in this region, the patterns probably reflect the change in the consumption habits of the population, with higher prevalence in men from lower social classes and an increase from 1960 onwards in the number of women smokers from higher social classes [28–30]. The positive social gradient observed in men reflects the higher prevalence of smokers in areas of higher deprivation [31,32], in addition to a more substantial reduction in smoking in areas of lower deprivation [33].

### Cancer of the colon, rectum and anus

Colorectal cancer mortality rate in Andalusia was 27.93 per 100,000 inhabitants in men and 13.32 per 100,000 for women. According to the Spanish Association Against Cancer (AECC), in 2017 Andalusia was the autonomous community with the highest incidence and mortality rates for this type of cancer [34].

The literature presents contradictory results regarding the association between colon and rectal cancer mortality and levels of deprivation. Colon and rectal cancer has been positively associated with high deprivation levels by some studies, and negatively by others, for both men and women [35].

One of the keys to reducing mortality due to colorectal cancer is early diagnosis, and there is a relationship between socio-economic level, sex and age; and levels of participation in screening programmes–men with the lowest socio-economic levels have the lowest screening participation levels [36]. According to the AECC, at present screening programmes reach only 3.74% of the at risk Andalusian population, while in other autonomous communities this figure is 100%. In this sense, the results of the study [37], suggest that the origin of the positive gradient for socio-economic level may be found either in the rates of early diagnosis in the most deprived areas or in the socio-economic differences regarding access to optimum treatment. It is possible that for this reason, and the low participation levels in screening programmes in Andalusia, our study does not show clear positive associations between the deprivation index and mortality rates for this type of cancer – with the exception of the positive gradient observed for men in Seville.

### Breast cancer

Although there has been a moderate overall decrease of 1.70 percentage points in breast cancer mortality rates in the last few years, breast cancer remains the principal cause of cancer-related deaths in Spanish women. The 2013 breast cancer mortality rate in Andalusia was higher than the Spanish national rate at 18.90 per 100,000 inhabitants [2].

As has also been noted by previous studies [38,39], there is a pattern of excess mortality that continues to affect the western provinces of Andalusia (Cadiz, Huelva and Seville). As with colorectal cancer, early diagnosis is one of the most important factors for reducing breast cancer mortality rates. Since 1990, there has been a gradual introduction of screening programmes for breast cancer across the Spanish autonomous communities. In Andalusia, the programme has been operating since 1995, with approximately 78% coverage of women between 50 and 69; this is within the objectives set out by the European Council Against Cancer, which recommends a minimum of 70% coverage. According to the Andalusian health survey data, the provinces with lowest coverage were Huelva (30.60%) and Seville (34.60%) in 2007, and Cadiz (40.70%) and Seville (43.70%) in 2011 [40], which suggests a possible explanation for the excess mortality highlighted in the western provinces by our study.

The literature provides numerous references showing the existence of social gradients for breast cancer, and associations similar to those we found for all of Andalusia have been observed [12,41]. In part, these can be attributed to the unequal variation of risk factors in the different deprivation levels [42]. Women with lower deprivation levels generally push back the age of first pregnancy, have fewer children, and different age patterns regarding the ages at which the first menstrual cycle occurs and at which the menopause begins [12,43], all of which are known risk factors for breast cancer.

In 2007, an inequality in take up of breast cancer screening according to social class was identified by the Andalusian health survey, with only 33.40% accessing screening in the lowest segment against 46.20% for those in less deprived groups. That said, in 2011 there were no observable differences according to deprivation level, and there was a notable increase in mammography take up for women with lower educational levels between 2007 (42%) and 2011 (56.50%) [40].

We hope that in the future the screening programme in Andalusia will succeed in reaching more at risk women than it does currently, and in doing so reduce breast cancer mortality as well as the interprovincial differences found in this study.

### Prostate cancer

In Andalusia the mortality rate for prostate cancer in 2013 was 15.43 per 100,000, which was a reduction of approximately 1.23% since 2003 [2]. From 1989 to 2008 prostate cancer mortality rates in Andalusia were lower than for Spain overall, disregarding the higher mortality in Cadiz province [25], which was also highlighted in our study. There is an inverse association between Type 2 diabetes mellitus or users of oral antidiabetic drugs and the risk of contracting prostate cancer [44,45]. The rates of diabetes mellitus mortality in Andalusia are higher than the national average [46], which, as a result of the protective effect of this condition, could be a possible explanation for the low prostate cancer mortality observable in Andalusia [3].

As yet there is little evidence regarding the potential relationship between deprivation levels and prostate cancer mortality. Some studies which used socioeconomic variables at an individual level did find evidence of positive social gradients in prostate cancer mortality [47,48]. However, our study did not provide conclusive results, possibly due to the utilization of data at census-tract rather than individual level.

### Bladder cancer

The bladder cancer mortality rate for Andalusia in 2013 was 13.31, higher than in the rest of Spain; however, it showed a downwards trend of 0.97% per year during the last few years [2]. The rate for women was 1.77 per 100,000. As regards the spatial distribution of mortality, we once again found a weak East-West pattern, with higher mortality in the provinces of Cadiz, Huelva and Seville. This result has already been highlighted by previous studies [24]. Various studies have associated bladder cancer with occupational exposure to chemicals [49]. For this reason, the provinces of Cadiz, Huelva and Seville have been studied as a result of their elevated levels of environmental contamination, with high concentrations of heavy metals found in waterway sediment across the area [50,51], which suggests that industrial activity in Cadiz, Huelva and Seville, as well as mining in the case of the latter two, could be associated with the pattern of excess mortality found in our study [52].

The association between deprivation levels and this kind of cancer has presented conflicting results, with only a few countries finding a relationship [53]. Across all of Andalusia, we found an increase in RRs for men in intermediate levels of deprivation, with a moderate increase in risk between the lowest and highest levels of deprivation. This is despite the known association between bladder cancer and smoking, and that there is a higher prevalence of smokers in groups with higher deprivation levels, especially in men. Previous studies have presented results that put the mortality rates for bladder cancer in Andalusia at comparable levels to other autonomous communities in Spain, despite the high proportion of smokers [54]. Our finding of higher RRs for intermediate levels of deprivation suggests that there are other risk factors at work, in addition to tobacco, which may be affecting the Andalusian population.

### Stomach cancer

The 2013 age-adjusted mortality rates for stomach cancer were 9.51 cases per 100,000 inhabitants for men and 4.11 per 100,000 for women. In Andalusia there was a trend of approximately a 2.50% reduction per year for both sexes [2]. Similar to the results our research obtained, many studies have found an association between stomach cancer and deprivation levels, showing higher mortality and incidence as well as lower survival rates for patients with higher levels of material deprivation [55,56].

To a large extent, this association can be explained by the presence of risk factors related to stomach cancer which are strongly associated with higher levels of deprivation: poor sanitary conditions that lead to H. pylori infection, higher prevalence of smokers, excessive alcohol consumption, as well as higher levels of processed food consumption and lower fruit and vegetable consumption [57].

### Strengths and limitations

Although the data source used for this study was longitudinal, the analysis was carried out using cross-sectional methodology, meaning that the ecological fallacy may be present, and which constitutes a limitation of this study. That said, small area studies tend to reduce the impact of the fallacy, as populations defined by administrative areas tend to be more homogeneous. Even so, although we can define the geographic distribution of mortality, we are unable to explain why exactly the risk is higher in particular areas, nor an association between mortality and the risk factor being studied. For this reason, ecological studies should always be accompanied by further research at an individual level.

By using census tracts, territorial units containing a similar population, the standardization of uncertainties in the estimation of mortality rates was facilitated; however, the actual area of census tracts varies considerably, as do their boundaries. The presence of few cases for particular types of cancer in some census tracts, or an excess of census tracts with null cases could cause changes in the probability distribution. Results obtained by other studies, however, suggest that the model used produces the results with best fit when analysing a single disease [58]. By the same token, this scarcity of cases means that the deprivation index used better reflects the differences that exist between different levels of deprivation in municipalities with large populations.

Although many different studies have already analysed the distribution of mortality in Spain against diverse causes at census-tract level [8,9,10], this is the first time that the BDLPA for all of Andalusia has been analysed by cause of death and census tract, as well as their association (s) with the level of deprivation of each census tract. As a result of the longitudinal nature of the data used, the study reduces classification bias as a result of changes to place of residence and/or migrations [59], as these were taken into account in our data source.

## 5. Conclusions

The geographical patterns of excess mortality that were observed in the years prior to our study in the western Andalusian provinces of Cadiz, Huelva and Seville continued for lung and bladder cancer in men, as well as for breast cancer in women. For Andalusia overall we found a positive social gradient for stomach and lung cancers in men, while for women there was a negative social gradient for lung cancer and a positive one for stomach cancer.

Knowledge regarding the spatial distribution of cancer mortality at small area level and the regional socioeconomic inequalities linked to this distribution should contribute to the design of social and health policies aimed at tackling cancer mortality and social inequality in those areas with excess mortality and/or higher levels of deprivation where specific local policies are required.

## References

[1] Instituto de Estadística y Cartografía de Andalucía. Estadística de Defunciones según la Causa de Muerte en el año 2013. http://www.ine.es/prensa/np896.pdf. Accessed 29 May 2018.

[2] López-Abente G, Núñez O, Pérez-Gómez B, Aragonés N, Pollán M. La situación del cáncer en España: Informe 2015. Madrid: Instituto de Salud Carlos III; 2015.

[3] López-Abente G, Ramis R, Pollán M, Aragonés N, Pérez-Gómez B, Gómez-Barroso D, et al Atlas Municipal de Mortalidad Por Cáncer En España, 1989–1998. Madrid: Instituto de Salud Carlos III; 2006.

[4] Costa-Font J & Gil J. Exploring the pathways of inequality in health, health care access and financing in decentralized Spain. J Eur Soc Policy. 2009;19(5):446–458.

[5] Cruces E, Haro J, Sarrión MD. Análisis estadístico de la realidad socioeconómica en Andalucía. Una aproximación a escala municipal. Investigaciones Regionales. 2009;18:107–138.

[6] Junta de Andalucía. II Plan Integral de Oncología de Andalucía. Análisis situación cáncer Andalucía 2007–2012. http://www.juntadeandalucia.es/salud/export/sites/csalud/galerias/documentos/c_1_c_6_planes_estrategias/plan_oncologia/02_analisis_de_situacion.pdf. Accessed 13 April 2018.

[7] Instituto de Estadística y Cartografía de Andalucía. Andalucía. Datos Básicos 2013.2013. http://www.juntadeandalucia.es/institutodeestadisticaycartografia/dtbas/dtb13/ADB2013.pdf. Accessed 13 February 2018.

[8] Borrell C, Marí-Dell’Olmo M, Serral G, Martínez-Beneito M, Gotsens M. Inequalities in mortality in small areas of eleven Spanish cities (the multicenter MEDEA project). Health Place. 2010;16(4):703–11. 10.1016/j.healthplace.2010.03.002 20399699

[9] Cambra K, Martinez-Rueda T, Alonso-Fustel E, Cirarda FB, Audicana C, Esnaola S, et al Association of proximity to polluting industries, deprivation and mortality in small areas of the Basque Country (Spain). Eur J Public Health. 2013;23(1):171–6. 10.1093/eurpub/ckr213 22315463

[10] Puigpinós-Riera R, Marí-Dell'Olmo M, Gotsens M, Cano-Serral G, Ascaso C, Borrell C, et al Cancer mortality inequalities in urban areas: a bayesian small area analysis in spanish cities. Int J Health Geogr. 2011;10:6 10.1186/1476-072X-10-6 21232096 PMC3033786

[11] Barceló MA, Saez M, Cano-Serral G, Martínez-Beneito MA, Martínez JM; Borrell C, et al Métodos para la suavización de indicadores de mortalidad: aplicación al análisis de desigualdades en mortalidad en ciudades del estado español (Proyecto MEDEA). Gac Sanit. 2008;22(6):596–608. 10.1016/s0213-9111(08)75362-7 19080940

[12] Fernandez E, Borrell C. Cancer mortality by educational level in the city of Barcelona. Brit J Cancer. 1999;79(3–4):684–689. 10.1038/sj.bjc.6690108 10027350 PMC2362440

[13] Instituto de Estadística y Cartografía de Andalucía. Junta de Andalucía. Estadísticas Longitudinales de Supervivencia y Longevidad en Andalucía, 2002–2013. https://www.ieca.juntaandalucia.es./longevidad/index.htm. Accessed 10 August 2017.

[14] Ruiz-Ramos M, Escolar-Pujolar A, Sánchez Perea J, Garrucho Rivero G. Evolución de las desigualdades sociales en la mortalidad general de la ciudad de Sevilla (1994–2002). Gac Sanit. 2006; 20(4), 303–10. 10.1157/13091146 16942718

[15] Bentley R, Kavanagh AM, Subramanian SV, Turrell G. Area disadvantage, individual socio-economic position, and premature cancer mortality in Australia 1998 to 2000: a multilevel analysis. Cancer Causes Control. 2008;19(2):183–193. 10.1007/s10552-007-9084-7 18027094

[16] UNSCEAR. UNSCEAR 2006 Report: Volume I e Annex A: Epidemiological Studies of Radiation and cancer. http://www.unscear.org/unscear/en/publications.html. Accessed 20 October 2019.

[17] Besag J, York J, Mollié A. Bayesian image restoration with two applications in spatial statistcs. Ann Inst Stat Math. 1991;43:1–59.

[18] Besag J. Spatial interaction and the statistical analysis of lattice systems. J R Stat Soc Series B Stat Methodol. 1974;36(2):192–225.

[19] Clayton D, Bernardinelli L, Montomoli C. Spatial correlation in ecological analysis. Int J Epidemiol. 1993;22(6):1193–202. 10.1093/ije/22.6.1193 8144305

[20] Spiegelhalter DJ, Best NG, Carlin BP, van der Linde A. Bayesian measures of model complexity and fit. J R Stat Soc Series B Stat Methodol. 2002;64:583–616.

[21] Rue H, Martino S., and Chopin N. Approximate Bayesian inference for latent Gaussian models using integrated nested Laplace approximations (with discussion). J R Stat Soc Series B Stat Methodol. 2009;71(2):319–392.

[22] The R-INLA project. http://www.r-inla.org/. Accessed 20 June 2017.

[23] R Development Core Team: R: A Language and Environment for Statistical Computing. Vienna, Austria: R Foundation for Statistical Computing; 2005.

[24] Monmonier M. How to lie with maps. 2nd ed. London: University of Chicago Press, UK; 1996.

[25] López-Abente G, Aragonés N, Pérez-Gómez B, Pollán M, García-Pérez J, Ramis R, et al Time trends in municipal distribution patterns of cancer mortality in Spain. BMC Cancer. 2014;14:535 10.1186/1471-2407-14-535 25060700 PMC4124154

[26] Antunes JL, Borrell C, Rodríguez-Sanz M, Pérez G, Biazevic MG, Wünsch-Filho V. Sex and socioeconomic inequalities of lung cancer mortality in Barcelona, Spain and São Paulo, Brazil. Eur J Cancer Prev. 2008;17:399–405. 10.1097/CEJ.0b013e3282f75f17 18714180

[27] Stewart BW, Kleihues P. World cancer report. Lyon: IARC Press; 2003.

[28] Cayuela A, Rodriguez-Dominguez S, Lopez-Campos JL, Vigil E, Otero R. Lung cancer mortality trends in Spain between 1980 and 2005. Arch Bronconeumol. 2008;44:70–74. 10.1016/s1579-2129(08)60012-9 18361872

[29] Cayuela A, Rodriguez-Dominguez S, Lopez-Campos JL, Vigil E. Lung cancer mortality trends by geographical area in Spanish women, 1980–2005. Int J Tuberc Lung Dis. 2008; 12:453–457. 18371274

[30] Schiaffino A, Fernandez E, Borrell C, Salto E, Garcia M, Borras JM. Gender and educational differences in smoking initiation rates in Spain from 1948 to 1992. Eur J Public Health. 2003;13:56–60. 10.1093/eurpub/13.1.56 12678315

[31] Borrell C, Rue M, Pasarín MI, Rohlfs I, Ferrando J, Fernandez E. Trends in social class inequalities in health status, health-related behaviors, and health services utilization in a southern European urban area (1983–1994). Prev Med. 2000;31:691–701. 10.1006/pmed.2000.0751 11133336

[32] Shohaimi S, Luben R, Wareham N, Day N, Bingham S, Welch A, et al Residential area deprivation predicts smoking habit independently of individual educational level and occupational social class: a cross sectional study in the Norfolk cohort of the European Investigation into Cancer (EPIC- Norfolk). J Epidemiol Community Health. 2003;57:270–276. 10.1136/jech.57.4.270 12646543 PMC1732421

[33] Schiaffino A, Fernandez E, Kunst A, Borrell C, Garcı´a M, Borras JM, et al Time trends and educational differences in the incidence of quitting smoking initiation in Spain (1965–2000). Prev Med. 2007;45:226–232. 10.1016/j.ypmed.2007.05.009 17604832

[34] Observatorio del Cáncer AECC. http://observatorio.aecc.es. Accessed 4 June 2018.

[35] Aarts MJ, Lemmens VEPP, Louwman MWJ, Kunst AE, Coebergh JWW. Socioeconomic status and changing inequalities in colorectal cancer? A review of the associations with risk, treatment and outcome. Eur J Cancer. 2010;46:2681–2695. 10.1016/j.ejca.2010.04.026 20570136

[36] Molina-Barceló A, Salas Trejo D, Peiró-Pérez R, Málaga López A. To participate or not? Giving voice to gender and socio-economic differences in colorectal cancer screening programmes. Eur J Cancer Care. 2011;20(5):669–78. 10.1111/j.1365-2354.2011.01263.x 21771129

[37] Nur U, Rachet B, Parmar MKB, Sydes MR, Cooper N, Lepage C,1,et al No socioeconomic inequalities in colorectal cancer survival within a randomised clinical trial. Br J Cancer. 2008;99:1923–8. 10.1038/sj.bjc.6604743 19034284 PMC2600684

[38] Ocaña-Riola R, Montaño-Remacha C, Mayoral-Cortés JM. Geographical and Temporal Variations in Female Breast Cancer Mortality in the Municipalities of Andalusia (Southern Spain). Int J Environ Res Public Health. 2016;13:1162. 10.3390/ijerph13111162 PMC5129372 27879690

[39] Pollán M, Ramis R, Aragonés N, Pérez-Gómez B, Gómez D, Lope V. Municipal distribution of breast cancer mortality among women in Spain. BMC Cancer. 2007;7:78 10.1186/1471-2407-7-78 17488519 PMC1872033

[40] Encuesta andaluza de salud 2011–2012. Muestra de adultos. https://www.juntadeandalucia.es/export/drupaljda/salud_5af9587a45705_EAS_2011_2012_Adultos.pdf. Accessed 1 September 2018.

[41] Lundqvist A, Andersson E, Ahlberg I, Nilbert M, Gerdtham U. Socioeconomic inequalities in breast cancer incidence and mortality in Europe-a systematic review and meta-analysis. Eur J Public Health. 2016;26(5):804–813. 10.1093/eurpub/ckw070 27221607 PMC5054273

[42] Heck KE, Wagener DK, Schatzkin A, Devesa SS, Breen N. Socioeconomic status and breast cancer mortality, 1989 through 1993: an analysis of education data from death certificates. Am J Public Health. 1997;87:1218–1222. 10.2105/ajph.87.7.1218 9240118 PMC1380902

[43] Kelsey JL, Horn-Ross PL. Breast cancer: magnitude of the problem and descriptive epidemiology. Epidemiol Rev. 1993;15:7–16. 10.1093/oxfordjournals.epirev.a036118 8405214

[44] Kasper JS, Giovannucci E. A meta-analysis of diabetes mellitus and the risk of prostate cancer. Cancer Epidemiol Biomarkers Prev. 2006;15:2056–62. 10.1158/1055-9965.EPI-06-0410 17119028

[45] Murtola TJ, Tammela TL, Lahtela J, Auvinen A. Antidiabetic medication and prostate cancer risk: a population-based case-control study. Am J Epidemiol. 2008;168:925–31. 10.1093/aje/kwn190 18700234

[46] Instituto Nacional de Estadística. Notas de prensa. Defunciones según la causa de muerte 2001; 2003.

[47] Tomic K, Ventimiglia E, Robinson D, Häggström C, Lambe M, Stattin P. Socioeconomic status and diagnosis, treatment, and mortality in men with prostate cancer. Nationwide population-based study. Int J Cancer. 2018;142:2478–2484. 10.1002/ijc.31272 29363113 PMC5947133

[48] Larsen SB, Brasso K, Christensen J, Johansen C, Tjønneland A, Friis S, et al Socioeconomic position and mortality among patients with prostate cancer: influence of mediating factors. Acta Oncol. 2017;56(4):563–568. 10.1080/0284186X.2016.1260771 27911129

[49] Gonzalez CA, Lopez-Abente G, Errezola M, Escolar A, Riboli E, Izarzugaza I, et al Occupation and bladder cancer in Spain: a multi-centre case-control study. Int J Epidemiol. 1989;18:569–577. 10.1093/ije/18.3.569 2681016

[50] CSIC (Consejo Superior de Investigaciones Científicas): Informes del estudio sobre el diagnóstico ambiental y sanitario de la ría de Huelva. Segundo informe del estudio que coordina el Consejo Superior de Investigaciones Científicas sobre el diagnóstico ambiental y sanitario de la ría de Huelva. 2005. http://www.csic.es/wi/informes_csic.jsp. Accessed 7 January 2018.

[51] Grupo de Trabajo de la Sociedad Española de Epidemiología: Dictamen realizado por encargo del Defensor del Pueblo Andaluz sobre "El exceso de mortalidad y morbilidad detectado en varias investigaciones en El Campo de Gibraltar". 2013. http://www.defensordelpuebloandaluz.es/informe-epidemiologico-campo-de-gibraltar. Accessed 13 December 2017.

[52] Lopez-Abente G, Aragones N, Ramis R, Hernandez-Barrera V, Perez-Gomez B, Escolar-Pujolar A, et al Municipal distribution of bladder cancer mortality in Spain: possible role of mining and industry. BMC Public Health. 2006;6:17 10.1186/1471-2458-6-17 16438735 PMC1409784

[53] Faggiano F, Partanen T, Kogevinas M, Boffetta P. Socioeconomic differences in cancer incidence and mortality. IARC Sci Publ. 1997;138: 65–176. 9353664

[54] Cózar JM, Miñana B, Palou-Redorta J, Medina RA, De la Rosa-Kehrmann F, Lozano-Palacio F, et al Análisis comparativo de la incidencia de cáncer de vejiga en las comunidades de Andalucía, Cataluña y Comunidad de Madrid en el año 2011. Actas Urol Esp. 2015;39:420–8. 10.1016/j.acuro.2014.11.003 25554606

[55] Nagel G, Linseisen J, Boshuisen HC, Pera G, Del Giudice G, Westert GP, et al Socioeconomic position and the risk of gastric and oesophageal cancer in the European Prospective Investigation into Cancer and Nutrition (EPIC-EURGAST). Int J Epidemiol. 2007;36:66–76. 10.1093/ije/dyl275 17227779

[56] Uthman OA, Jadidi E, Moradi T. Socioeconomic position and incidence of gastric cancer: a systematic review and meta-analysis. J Epidemiol Community Health. 2013; 67:854–860. 10.1136/jech-2012-201108 23929615

[57] Shibata A, Parsonnet J. Stomach Cancer In: Schottenfeld D, Fraumeni JF Jr, editors. Cancer Epidemiology and Prevention. New York: Oxford University Press; 2006 P. 707–20.

[58] Best N, Richardson S, Thomson A. A comparison of Bayesian spatial models for disease mapping. Stat Met Med Res. 2005;14:35–9. 10.1191/0962280205sm388oa 15690999

[59] Ocaña-Riola R, Fernández A, Mayoral JM, Toro S, Sánchez- Cantalejo C. Uncontrolled migrations as a cause of inequality in health and mortality in small area studies. Epidemiology. 2009;20:411–418. 10.1097/EDE.0b013e318196aaf4 19289967

